# The Distribution of IgT mRNA^+^ Cells in the Gut of the Atlantic Salmon (*Salmo salar* L.)

**DOI:** 10.3390/ani13203191

**Published:** 2023-10-12

**Authors:** Pedro Luis Castro, Fran Barac, Tom Johnny Hansen, Per Gunnar Fjelldal, Ivar Hordvik, Håvard Bjørgen, Erling Olaf Koppang

**Affiliations:** 1 GIA-ECOAQUA, Universidad de Las Palmas de Gran Canaria, 35001 Telde, Spain; 2 Unit of Anatomy, Veterinary Faculty, Norwegian University of Life Sciences, 1433 Ås, Norway; fran.barac@nmbu.no (F.B.); havard.bjorgen@nmbu.no (H.B.); erling.o.koppang@nmbu.no (E.O.K.); 3 Matre Research Station, Institute of Marine Research, 5984 Matredal, Norway; tomh@hi.no (T.J.H.); pergf@hi.no (P.G.F.); 4 Institute of Biology, University of Bergen, 5007 Bergen, Norway; ivar.hordvik@uib.no

**Keywords:** Atlantic salmon, gut, IgT, pIgR, in situ hybridization, mucosal immunity, vaccination

## Abstract

**Simple Summary:**

IgT is a specific type of antibody found in teleost fish that is crucial for protecting mucosal surfaces. However, studying IgT is challenging due to the limited available cell markers. Here, we investigated the distribution of IgT mRNA^+^ cells and pIgR mRNA^+^ cells (essential for Ig transport over cell membranes) in Atlantic salmon (*Salmo salar*) intestines. Using in situ hybridization, we examined two different sizes (developmental stages) of the fish and compared the hybridization-positive cell distribution between vaccinated and unvaccinated fish. Our findings revealed that IgT mRNA^+^ cells were mainly located beneath the intestinal mucosa, specifically in the *lamina propria*, while pIgR mRNA^+^ cells were found in both the *lamina propria* and mucosa. Additionally, vaccinated fish exhibited abdominal adhesions with tissue containing IgT and pIgR mRNA^+^ cells. We observed regional variations in the distribution of IgT mRNA^+^ cells within the salmon intestine that were unaffected by intraperitoneal vaccination but sensitive to fish age. This study provides new insights into the distribution and dynamics of IgT and pIgR mRNA^+^ cells, advancing our understanding of the spatial mucosal immune system and its implications for teleosts, with potential applications in aquaculture.

**Abstract:**

The newly discovered IgT^+^ B cell is thought to play a dominant role in mucosal immunity, but limited studies have examined its distribution in fish species, hindering our understanding of its function. This study investigated IgT and poly Ig receptor (pIgR) mRNA^+^ cell distribution in Atlantic salmon (*Salmo salar*) gut using RNAscope in situ hybridization (ISH) and assessed the effects of vaccination. The pyloric caeca, mid-intestine (first and second parts), and posterior segment in two weight stages (Group 1: avg. 153 g, Group 2: avg. 1717 g) were examined in both vaccinated and unvaccinated fish. ISH revealed more IgT mRNA^+^ cells in the second part of the midgut compared to other intestinal segments, as well as a higher number of positive cells in Group 2 (older fish). In line with previous findings, intraperitoneal vaccination had no significant impact on the number of IgT^+^ transcripts. IgT mRNA^+^ cells were found mostly in the *lamina propria* and near capillaries, while pIgR was registered in both the *lamina propria* and mucosa. Interestingly, vaccinated fish presented adhesions and granulomatous tissue in the peritoneum, with both IgT and pIgR mRNA^+^ cells. Taken together, these results suggest that the distribution of IgT mRNA^+^ cells in the intestine of Atlantic salmon is region-specific and is not affected by intraperitoneal vaccination but varies with fish age.

## 1. Introduction

Three isotypes of immunoglobulins (Ig) have been identified in teleost fish, including IgM, IgD, and IgT, also called IgZ in some species [1]. While the IgM antibody class is recognized for being involved both in systemic and local responses, IgT, a teleost-specific class, is considered to specialize in mucosal protection, thought to be equivalent to IgA in mammals [1,2,3]. IgT has been demonstrated to participate in immune responses against parasites [4,5], viruses [6,7], and bacteria, and also to respond to DNA vaccination [6,8]. In most of these studies, IgT has been pointed out as a key mucosal immunoglobulin. For fishes, the gut constitutes a large body area that is in constant contact with pathogens and plays a vital role in immune defense against inflammation and pathogen infection [9].

The teleost fish intestine includes anatomically defined segments with distinct physiological roles and immunological behavior. When comparing the different segments, the lymphoid cell content has been shown to differ [10,11]. This variation may also be observed in IgT expression in rainbow trout (*Oncorhynchus mykiss*) [12], in European seabass (*Dicentrarchux labrax*) [6], and in Atlantic salmon (*Salmo salar*) [13]. Transcriptional investigations dominate the published literature regarding fish intestinal leukocyte content. Morphological studies are scarce, but reports show scattered lymphocytes in the *lamina propria* (LP) or between epithelial cells. Germinal centers, Peyer’s patches, and lymph nodes have not been reported in fish [6,14].

Even though IgT has been considered equivalent to IgA in mammals and birds, this view could be nuanced, as IgT presents unique traits depending on the species in which it is found. Progress within this field has been hampered by the general lack of cell markers targeting different immune molecules [15]. Research on different teleost Ig isotypes has mainly relied on gene expression studies. However, in several studies, especially those concentrating on IgM, multiple reliable antibodies have been employed to determine the serum titers [16,17]. Thus, morphological studies on IgT and IgT^+^ B cells are limited to only a few fish species. The application of improved morphological techniques, particularly in situ hybridization (ISH), enables research combining molecular biology with morphological investigations [14]. In 2005, IgT/IgZ was first discovered in rainbow trout and zebrafish (*Danio rerio*) [18,19]. To date, this teleost Ig isotype’s immunological properties and biological functions have been addressed primarily in rainbow trout [4]. Accordingly, research on the immune systems of other species is attracting increased attention. Atlantic salmon is a species of significant economic importance [20] and, in addition, presents a unique model species, with both freshwater and saltwater as natural habitats. A large diversity of antigen receptors in Atlantic salmon may have evolved to protect against various pathogens [21]. Currently, no reports address the morphological occurrence of IgT^+^ cells in the Atlantic salmon intestine or the possible impact of vaccination.

The polymeric immunoglobulin receptor (pIgR) is a key player during mucosal immune responses [22]. In fish, pIgR can bind to IgT and/or IgM [23]. However, little is known about pIgR-mediated immune excretion. Through transcytosis, the pIgR could transport mucosal Ig complexes from the LP across the intestinal epithelium into the gut mucus [24].

The present study sought to precisely determine the spatial localization and distribution of IgT mRNA^+^ cells within various regions of the Atlantic salmon intestine using in situ hybridization. Additionally, we conducted a complementary assessment of pIgR mRNA^+^ cells in the second segment of the midgut to elucidate potential pathways for Ig molecule transportation to the intestinal lumen. Finally, we investigated the impact of conventional intraperitoneal vaccination on the presence and dispersion of these cells. In addition, we investigated how traditional intraperitoneal vaccination affected the appearance and distribution of these cells. Here, we show differences in distribution related to the intestinal compartments and, furthermore, that maturation impacts the appearance of IgT-expressing cells, while there are no recordable effects following intraperitoneal vaccination.

## 2. Materials and Methods

### 2.1. Animal Study

Atlantic salmon from the Aquagen strain were kept in indoor tanks at the Matre Research Station (Institute of Marine Research, Matre, Norway), with a seawater temperature of 9 °C, a simulated natural photoperiod, and a tank size of 1.5 × 1.5 m × 0.7 m in depth. The fish were transferred to seawater at a time that corresponded to the natural life cycle and were fed on a standard commercial diet up until sampling. The fish were held in optimal water conditions, ocean water was taken from a depth of 90 m, and the oxygen saturation was 100% in the inlet flow and 80% in the outlet flow. The test population was divided into 2 groups. One half was vaccinated intraperitoneally with an inactivated, multivalent vaccine against furunculosis, classical vibriosis, cold water vibriosis, wound disease, and infectious pancreatic necrosis (IPN) (Norvax^®^ Minova 6, Boxmeer, The Netherlands), and the other half was left unvaccinated. The fish were weighed 7 days after vaccination, weighing an average of 58 g. Vaccinated and non-vaccinated fish were reared in different tanks. After 4 months and 13 months post-vaccination (average weight 153 g (Group 1) and 1717 g (Group 2), respectively), the fish were euthanized with an overdose of Finquel 0.5 g/L, then bled out and sampled. At both sampling points, organ segments from 3 unvaccinated and 3 vaccinated Atlantic salmon were collected; *n* = 12 (Table 1).

### 2.2. Sampling

The anatomical nomenclature suggested by Løkka et al. (2013) [25] was followed during sampling and processing. Thus, the pyloric caeca, first and second segments of the mid-intestine, and posterior segment were collected and fixed in formalin (4% formalin, 0.08 M sodium phosphate, pH 7.0), processed in a Thermo Scientific Excelsior^®^ tissue processor (Thermo Fisher Scientific, Waltham, MA, USA), and embedded in paraffin Histowax using a Tissue-Tek^®^ TEC 5 (Sakura Finetek, Alphen aan den Rijn, The Netherlands).

### 2.3. In Situ Hybridization

All fish from both groups were subjected to ISH, targeting IgT mRNA labeling [26]. For ISH aimed at pIgR, only the most relevant sections were included, based on the results for IgT mRNA, focusing on the second segment of the mid-intestine. The ISH procedure was performed using the RNAscope^®^ 2.5 HD Assay RED (Advanced Cell Diagnostics (ACD), Newark, CA, USA). The manufacturer designed and produced the probes, based on the provided IgT and pIgR sequences of Atlantic salmon (Table 2). In short, paraffin-embedded tissue sections (5 µm) were mounted on positively charged glass slides (Superfrost, Mentzel), dried at 37 °C for 48 h, and further incubated at 60 °C for 90 min. Subsequently, the samples were deparaffinized and treated for endogenous peroxidase blocking (10 min at RT), followed by target retrieval (15 min at 100 °C) and protease digestion (30 min at 40 °C) to enable the permeabilization of cells. For probe hybridization, the samples were incubated with the RNA scope probe for 2 h at 40 °C. A series of hybridizations were performed using different incubation times, according to the manufacturer’s instructions [27], to allow amplification of the signal. For signal detection, samples were treated with the Fast Red chromogenic substrate for 10 min and stained with a 50% Gill’s haematoxylin solution for 2 min. Samples were then dehydrated and mounted with EcoMount (BioCare Medical, Pacheco, CA, USA). The head kidney from vaccinated and unvaccinated Atlantic salmon was used as a positive control for both probes (the head kidney of Atlantic salmon is a B-cell-containing immune structure). A probe targeting Peptidylpropyl Isomerase B (*ppib*) in Atlantic salmon (Advanced Cell Diagnostics) was used as reference target gene expression to test for RNA integrity in the samples. Dihydrodipicolinate reductase (*dapB*), a bacterial control gene from *Bacillus subtilis* (Advanced Cell Diagnostics), was used as the negative control gene to confirm the absence of the background and non-specific cross-reactivity of the assay (Table 2, Figure S1 in the Supplementary Materials).

### 2.4. Image Acquisition and mRNA^+^ Cell Counting

**Figure 1:**
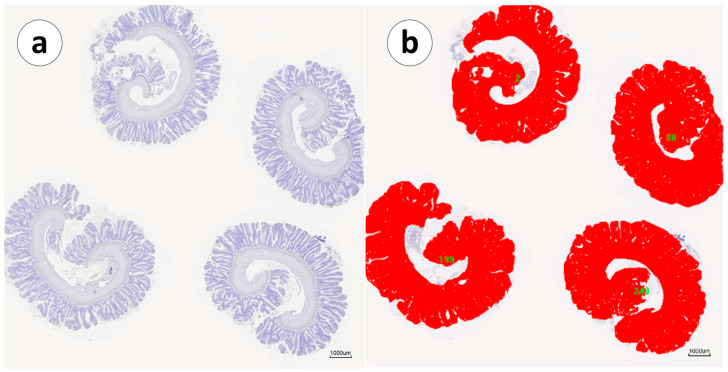
(**a**) Scanned section with samples from the second segment of the mid-intestine of Atlantic salmon gut (Group 2). (**b**) Attached comparative image of the selected area graph (red). Scale bar: 100 µm.

The sections were scanned with a MoticEasyScan Pro digital scanner (Motic, Xiamen, China) operated using the Motic DS Assistant software (Motic VM V1 Viewer 2.0). Those figures framed with a blue line were digitalized with a Philips IntelliSite UltraFast Scanner system (Philips Digital Pathology Solutions, Best, The Netherlands) operated using the Philips IMS viewer system (Philips Digital Pathology Solutions). The intestine area was measured using the analySIS^®^ software package for Windows (Image Pro Plus^®^ V. 4.5.0.29) (Media Cybernetics, Silver Spring, MD, USA). Since the samples were cut in such a manner that there were multiple tissue sections per slide (4), the counting was conducted in all the sections of the gut that were present on each slide. Each gut section was manually selected with the eyedropper tool, converted into a binary format (Figure 1), and automatically measured after calibration with the scale bar.

The screening for positive signals included the whole section from the mucosa to the adventitia. IgT mRNA^+^ cell counting was performed using the analySIS^®^ software package, based on signal intensity in the corresponding channel. Subsequently, the measurements were converted to cell densities (mean number of positive cells per mm^2^). For pIgR mRNA^+^, due to the small and diffuse positivity in the tissue, a blinded semi-quantitative scoring was established, with the general immunopositivity distribution in each section (Table 3) being described by 2 trained independent blind observers. Similarly, the identical panel was monitored for IgT mRNA^+^ in the peritoneum due to the heterogeneity of the peritoneal tissue, which is unsystematically present and shows a variable occurrence of adipose tissue. The immunolabelling intensity grades were scored based on the average number of cells or dots as follows: + (weak), ++ (moderate), and +++ (strong) (see Table 3).

### 2.5. Statistical Analyses

The average of the 4 tissue sections on each slide and the average of the 3 fish studied according to treatment (size and immune status) were calculated (*n* = 12). Data were analyzed with IBM SPSS Statistics for Windows, Version 26.0 (Armonk, NY, USA: IBM Corp.), testing for normality and homogeneity of variance. The test results indicated that the data were non-normally distributed and a non-parametric Kruskal–Wallis test was performed. The significance was established, where *p* = 0.05.

### 2.6. Ethical Statement

All wet laboratory samplings were performed at the Institute of Marine Research, Matre Research Station (60° N, 5° E, Matredal, Western Norway), which is authorized for animal experimentation (Norwegian Food Safety Authority, facility 110), following international guidelines. The experiment and euthanasia procedure were approved (FOTS ID 5290) and conducted following the regulations. The fish were killed solely to use their tissues for research and were not exposed to pain or distress.

## 3. Results

**Figure 2:**
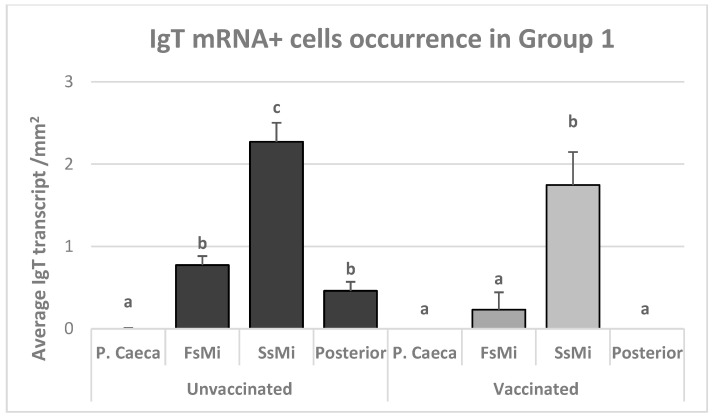
The mean number of IgT-transcript-positive cells in vaccinated and unvaccinated Atlantic salmon from Group 1in the different gut segments. Lowercase letters indicate significant differences (*p* < 0.05). Bars with the same letters are not significantly different via nonparametric analysis (Kruskal–Wallis ANOVA and multiple comparisons); *n* = 12.

**Figure 3:**
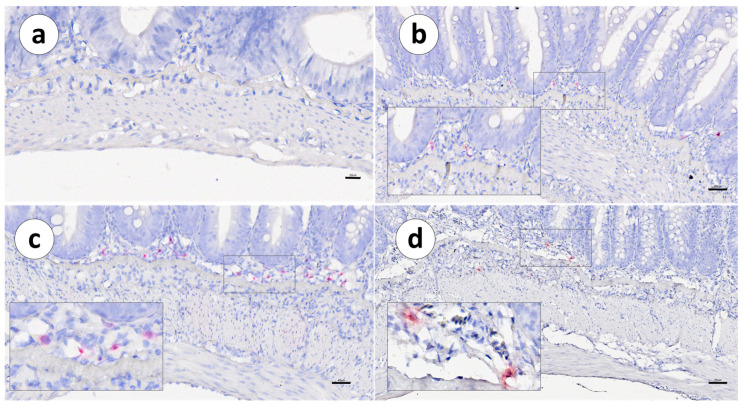
Group 1: RNAscope in situ hybridization, demonstrating the IgT mRNA distribution (red signal) in unvaccinated Atlantic salmon. (**a**) Pyloric caeca, (**b**) first segment of the mid-intestine, (**c**) second segment of the mid-intestine, and (**d**) posterior segment. Scale bar as follows: (**a**) 20 µm, (**b**) 40 µm, (**c**) 50 µm, and (**d**) 70 µm. Details: 8–10 µm.

**Figure 4:**
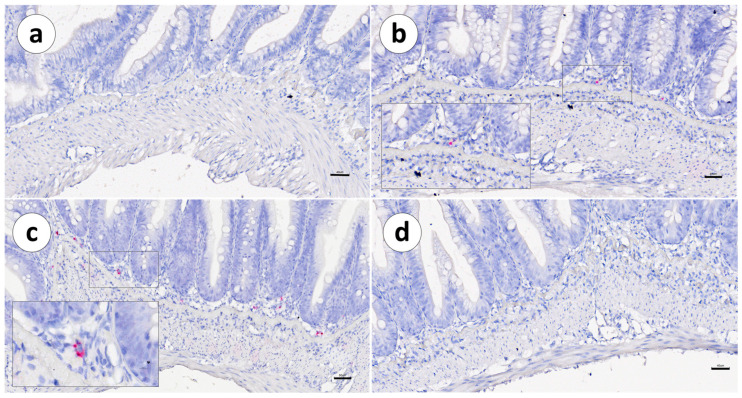
Group 1: RNAscope in situ hybridization, demonstrating the IgT mRNA distribution in vaccinated Atlantic salmon. (**a**) Pyloric caeca, (**b**) first segment of the mid-intestine, (**c**) second segment of the mid-intestine, and (**d**) posterior segment. Scale bar as follows: (**a**) 40 µm, (**b**) 40 µm, (**c**) 50 µm, and (**d**) 40 µm. Details: 8–10 µm.

In Group 1, IgT mRNA^+^ cells (red signal) were significantly higher in the second segment of the mid-intestine (Figure 2), compared with the other intestinal segments (Figure 3), in both unvaccinated (*p* ≤ 0.03) and vaccinated (*p* ≤ 0.04) groups (Figure 4).

There were no significant differences between the different intestinal segments when comparing the vaccinated group with the unvaccinated group (*p* > 0.05).

**Figure 5:**
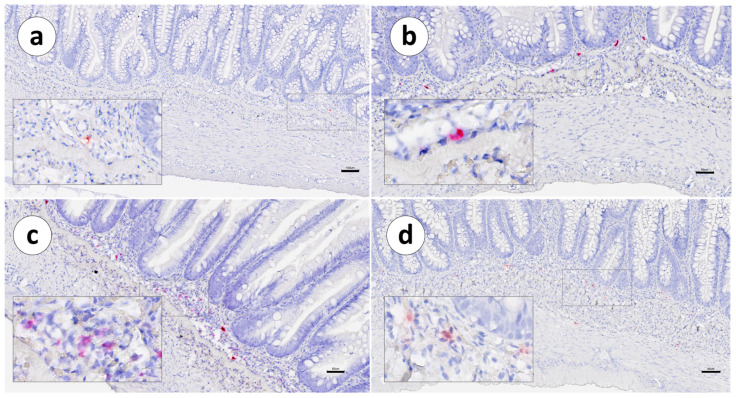
Groups 1 and 2: RNAscope in situ hybridization demonstrating the IgT mRNA distribution in the second segment of the mid-intestine of vaccinated and unvaccinated Atlantic salmon. (**a**) Group 1, unvaccinated, (**b**) Group 1, vaccinated, (**c**) Group 2, unvaccinated, and (**d**) Group 2, vaccinated. Scale bar as follows: (**a**) 30 µm, (**b**) 30 µm, (**c**) 100 µm, and (**d**) 100 µm. Details: 8–10 µm.

In both vaccinated and unvaccinated fish, the distribution of IgT mRNA^+^ cells was mainly restricted to the *lamina propria*, between the *stratum compactum* and the mucosa. Positive cells were not detected within the intestinal epithelium (Figure 3, Figure 4 and Figure 5a,b).

**Figure 6:**
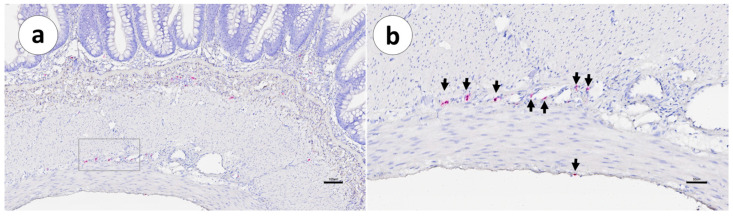
Group 1: RNAscope in situ hybridization, demonstrating the IgT mRNA distribution in the myenteric plexus of unvaccinated Atlantic salmon. (**a**) IgT-transcript-positive cells in relation to the blood vessel; (**b**) detail (arrows). Scale bar as follows: (**a**) 100 µm; (**b**) 50 µm.

The sporadic presence of IgT mRNA^+^ cells was noted in the *stratum granulosum* or in the myenteric plexus associated with small or medium-sized arterioles (Figure 6).

**Figure 7:**
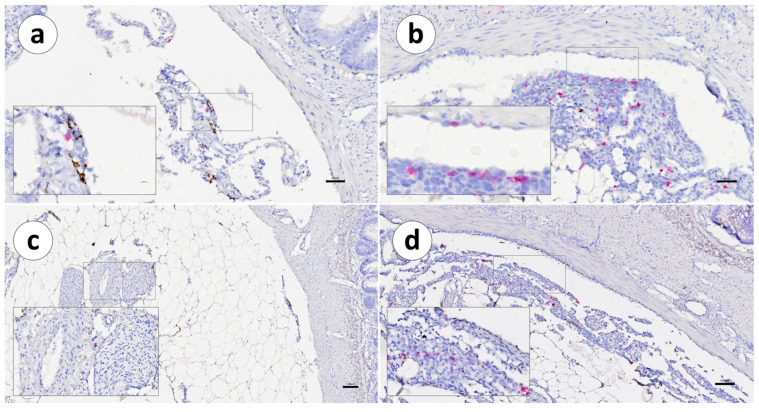
Groups 1 and 2: RNAscope in situ hybridization, demonstrating the IgT mRNA distribution in the peritoneal tissue of the second segment of the mid-intestine sections. (**a**) Group 1, unvaccinated, (**b**) Group 1, vaccinated, (**c**) Group 2, unvaccinated, and (**d**) Group 2, vaccinated. Scale bar as follows: (**a**,**b**) 40 µm; (**c**,**d**) 100 µm. Details: 8–10 µm.

In the visceral peritoneum (Table 3), positivity was mainly confined to the portion associated with the second segment of the mid-intestine in both unvaccinated and vaccinated fish (Figure 7).

In the sections from the vaccinated fish (Figure 7b,d), there were sporadic cellular inflammatory reactions, organized as nascent granuloma formations with macrophage-like and epithelioid cells and lymphocytes. In addition, there were some positive IgT mRNA^+^ cells in the peritoneum (Figure 7b). In these sections, the mesothelial cells of the peritoneum showed a cuboidal or cylindrical shape (Figure 7b).

**Figure 8:**
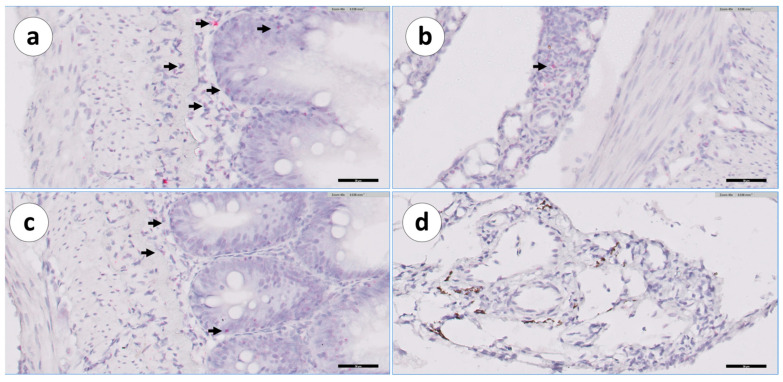
Group 1: RNAscope in situ hybridization, demonstrating the pIgR mRNA distribution in the second segment of the mid-intestine of Atlantic salmon. (**a**) Unvaccinated mucosa, (**b**) unvaccinated peritoneum, (**c**) vaccinated mucosa, and (**d**) vaccinated peritoneum. Arrows indicate pIgR-positive cells. Scale bars: 50 µm.

pIgR mRNA^+^ cells in the second segment of the mid-intestine from Group 1 (Table 3) were mainly distributed in the LP and the mucosa (Figure 8a) and in the peritoneum or adherences when present (Figure 8b).

pIgR mRNA^+^ cells were frequently found in association with the *tunica intima* of blood vessels (Supplementary Material Figure S2).

**Figure 9:**
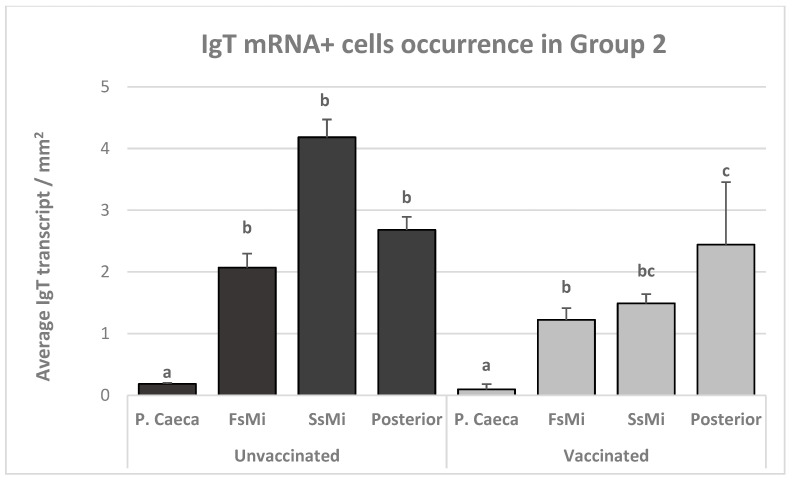
Group 2: mean number of IgT-transcript-positive cells of vaccinated and unvaccinated fish in different gut segments. Lowercase letters indicate significant differences (*p* < 0.05) between the groups. Bars with the same letters are not significantly different via non-parametric analysis (Kruskal–Wallis ANOVA and multiple comparisons) *n* = 12.

In Group 2, the number of IgT mRNA^+^ cells in the gut of the unvaccinated fish gradually increased from the pyloric caeca along the intestinal length, with the highest amount in the second segment of the mid-intestine (Figure 9).

**Figure 10:**
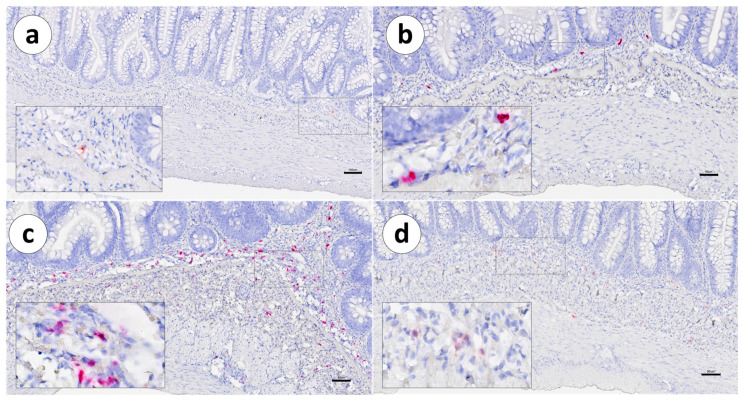
Group 2: RNAscope in situ hybridization, demonstrating IgT-transcripts in unvaccinated Atlantic salmon. (**a**) Pyloric caeca, (**b**) first segment of the mid-intestine, (**c**) second segment of the mid-intestine, and (**d**) posterior segment. Scale bar as follows: (**a**) 100 µm, (**b**) 60 µm, (**c**) 60 µm, and (**d**) 90 µm. Details: 8–10 µm.

In the pyloric caeca, as in Group 1, the number of positive cells was significantly lower compared to other gut segments (*p* ≤ 0.02; Figure 10).

**Figure 11:**
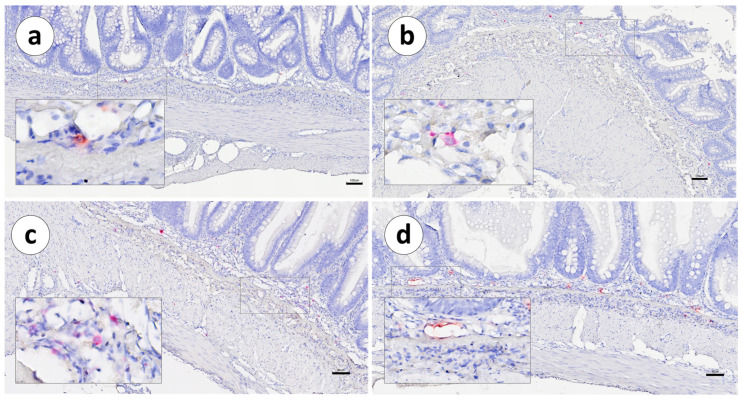
Group 2: RNAscope in situ hybridization, demonstrating IgT-transcripts in vaccinated Atlantic salmon. (**a**) Pyloric caeca, (**b**) first segment of the mid-intestine, (**c**) second segment of the mid-intestine, and (**d**) posterior segment. Scale bar as follows: (**a**) 70 µm, (**b**) 100 µm, (**c**) 80 µm, and (**d**) 80 µm. Details: 8–10 µm.

The differences in the last three segments were not significant when compared with each other (*p* ≥ 0.05). Similarly, in the vaccinated fish (Figure 9), the number of IgT mRNA^+^ cells was significantly lower in the pyloric caeca when compared to any other section (*p* ≤ 0.04; Figure 11).

**Figure 12:**
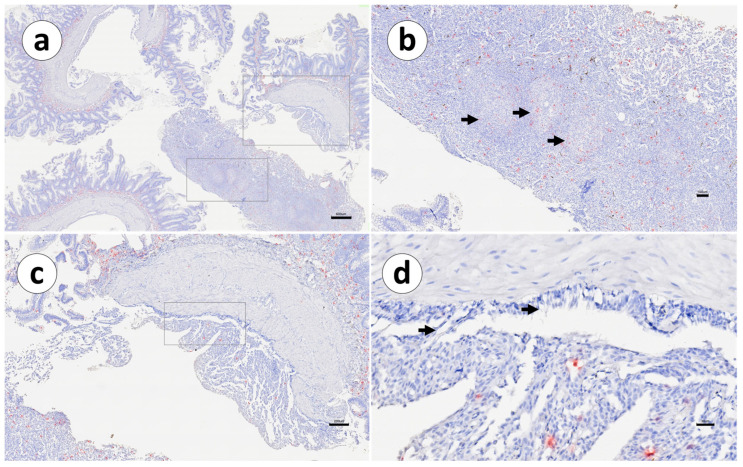
Group 2: RNAscope in situ hybridization, demonstrating IgT-transcripts in the posterior segment from a vaccinated Atlantic salmon. (**a**) General view, (**b**) granulomatous aggregates in the adherences, (**c**) detail of the adhesion, and (**d**) active mesothelium. Scale bar as follows: (**a**) 600 µm, (**b**) 100 µm, (**c**) 200 µm, and (**d**) 30 µm.

In addition, one individual in Group 2 presented an elevated number of IgT mRNA^+^ cells in the posterior segment, compared to other individuals from the same group (Figure 12).

The distribution of IgT mRNA^+^ cells was also preferentially confined to the LP (Figure 10 and Figure 11), as in Group 1. The sporadic presence of positive cells was noted outside the LP and was associated with small- or medium-sized arterioles (Figure 11a,d).

**Figure 13:**
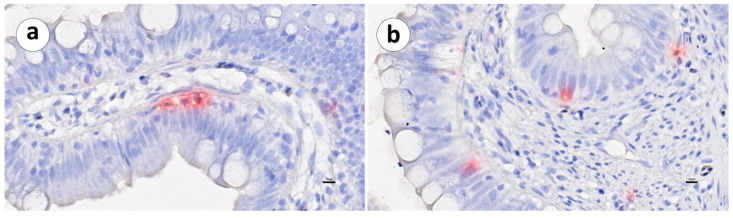
Group 2: RNAscope in situ hybridization, demonstrating IgT-transcripts in the mucosa of a posterior segment section from a vaccinated Atlantic salmon. (**a**) Posterior intestine mucosa detail; (**b**) posterior intestine mucosa detail. Scale bar: 10 µm.

Within the mucosal epithelial layer, we observed the presence of other cells intermingled with the epithelial cells (Figure 13), raising the possibility that these cells could represent intraepithelial lymphocytes (IELs).

The positive cells were small and spherical and were located above the basal lamina. IgT mRNA^+^ cells in the vaccinated group were found in the visceral layer of the peritoneum, especially in those areas close to the adherences. In the adherences’ surrounding areas, the serosa’s mesothelial cells also showed a modified shape, changing from a squamous to a cubic appearance (Figure 7d). In the visceral peritoneum (Table 3) of the second segment of the mid-intestine, unvaccinated fish displayed a few IgT mRNA^+^ cells (Figure 7c,d); this was not observed in the other portions. However, in the peritoneal adherences of the vaccinated fish, positive transcripts were present from the pyloric caeca through to the posterior segment, with a more prominent presence in the second segment of the mid-intestine (Figure 7d) and in the posterior segment sections (Figure 12d). The organization of the peritoneal adherences seemed similar to those found in Group 1, with different severity levels of intra-abdominal changes observed as granulomatous reactions, which included fibroblasts, macrophage-like cells, epithelioid cells, and melano-macrophages.

**Figure 14:**
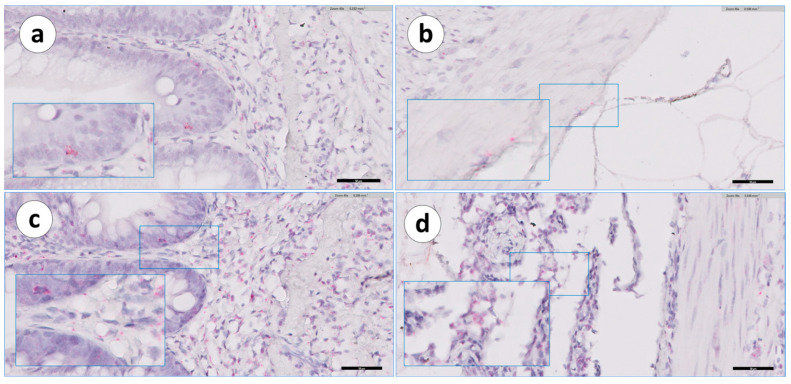
Group 2: RNAscope in situ hybridization, demonstrating the pIgR mRNA distribution in the second segment of the mid-intestine of Atlantic salmon: (**a**) unvaccinated second segment of the mid-intestine, (**b**) unvaccinated second segment of the mid-intestine peritoneum, (**c**) vaccinated second segment of the mid-intestine, and (**d**) vaccinated second segment of the mid-intestine peritoneum. Arrows indicate pIgR^+^ cells. Scale bar: 50 µm.

In the peritoneum, when comparing Groups 1 and 2, our results also revealed that the number of IgT^+^ transcripts was visibly higher in all evaluated intestine segments in Group 2 compared to Group 1 (Table 3). The pIgR mRNA^+^ cells in Group 2 (Table 3), as in Group 1, were identified in the *lamina propria* and the gut mucosa, interspersed between intestinal epithelial enterocytes (Figure 14).

The richest layers in terms of pIgR mRNA^+^ transcripts were the *lamina propria* and mucosa. pIgR transcript-positive cells were also present in the mesothelium and in the blood vessels but were less abundant in the muscular layer. The pIgR mRNA^+^ cell distribution in the peritoneum was more prominent when granulomatous tissue was recorded (Figure 14b,d).

## 4. Discussion

In this study, the distribution of IgT^+^ B cells throughout the intestine of Atlantic salmon was examined using ISH. Our results demonstrate variations in the incidence of IgT mRNA^+^ signals across different gut segments, these being most abundant in the second segment of the mid-intestine. Several other studies on Atlantic salmon have also pointed out that the second segment of the mid-intestine appears more immunologically active than any other area of the gastrointestinal tract [28,29]. The first systematic reference to this regionalization in Atlantic salmon was described by Løkka et al. in 2014 [13], wherein significantly higher transcript levels of the immunity-related genes MHC class II, sIgM, mIgM, and IgT were observed in the second segment of the mid-intestine and posterior segments compared with the pyloric caeca. In sea bass (*Dicentrarchus labrax*) and ballan wrasse (*Labrus bergylta*), differential expression along the intestinal tract was also described with ISH and RT-qPCR, with significant expression in the SsMi [7,30]. In rainbow trout gut, IgT^+^ B cell distribution was also found to be regionalized, showing the significant recruitment of B cells in the pyloric caeca after oral vaccination [12]. Our research reaffirms that the distribution of IgT^+^ B cells in the intestine of Atlantic salmon, as confirmed in various other fish species, exhibits regionalization, with varying levels of immune activity in different gut segments. The observed regionalization of IgT^+^ B cells in the intestine of Atlantic salmon underscores the complex relationship between immune responses and gut physiology.

Functional characteristics may explain the regional IgT differences observed in our study. Fuglem et al. (2010) [31] exposed different intestinal segments from salmonid fish to gold-BSA to identify antigen-sampling cells. Gold-bovine serum albumin uptake was restricted to a limited number of epithelial cells that were located in the mucosal folds in the second segment of the mid-intestine. These cells also showed some of the properties of microfold cells (M cells) in their lectin-binding capacity. By using transcriptome analysis, Atlantic salmon parr showed distinct responses in the midgut and hindgut (from the first segment of the mid-intestine to the posterior segment) under stress conditions derived from handling or temperature changes. In addition, dietary-dependent inflamed intestines, including dislocated epithelial cells in Atlantic salmon, have mainly been observed in the second segment of the mid-intestine [11,29]. In this segment, the wall is thinner than in the first segment, and this thin wall and the characteristic extensive folding (complex folds) suggest that little mechanical processing takes place in this region [11,25]. This segment seems to be especially important in antigenic uptake and corresponds with the intestinal region described in zebrafish exhibiting probable antigen-presenting enterocytes [32]. It is challenging to elucidate why the second segment of the mid-intestine appears more immunologically active in Atlantic salmon than other intestinal portions.

Fish size has been considered relevant to immune system functioning. Younger fish have been shown to exhibit significantly lower antibody levels, indicating that juvenile fish may not be able to mount as effective an immune response as young adult fish [33], which is especially relevant for IgM [34,35]. However, species-dependent differences are also relevant. For example, IgM concentration in the serum of Atlantic cod (*Gadus morhua*) continues to increase relative to the fish size, while in salmonids, the concentration progressively increases but is stabilized by sexual maturity [36]. The more significant amount of IgT mRNA^+^ cells found in all segments of older Atlantic salmon from Group 2 could be due to the developing immune systems of younger animals. Scarce studies have focused on how the size of the fish can influence the function of the immune system [31]. Thus, our results provide further insight into the impact of age on the presence of IgT^+^ B cells. It has been suggested that similarly to IgA and IgM in mammals, the transportation of polymeric IgT and IgM in teleost fish is mediated by the transmembrane transporter polymeric immunoglobulin receptor (pIgR) [2,37]. pIgR can transport pIg from the basolateral surface onto the apical surface of epithelial cells by transcytosis [38]. However, it has been demonstrated in rodents that only a small proportion of the IgA synthesized in the intestinal LP enters the gut lumen directly. Most of it travels through the mesenteric lymph, then into the bloodstream, and is finally transported into bile by hepatocytes to protect the intestinal epithelium [39]. The result of the present experiment agrees with this perception since the dispersed distribution of IgT^+^ cells, mainly confined to the LP and only rarely in the mucosa, does not favor IgT transepithelial transport. The upregulation of pIgR expression is an accepted phenomenon in mammals, one that seems to be driven by infection and inflammatory mediators [34]. It can also be downregulated, for instance, in the case of inflammatory bowel disease [40]. The local immune response involving pIgR and IgT has yet to be studied extensively in fish. In ballan wrasse, pIgR mRNA was significantly lower in the foregut and hindgut [25], where pIgR^+^ cells were detected in the LP under the basal lamina. In the present study, the location was similar. However, we also found an ample presence of pIgR in the peritoneal adherences and in the granulomatous tissue, which may indicate that vaccination can lead to the local production of Ig, which is then released into the bloodstream, or that there is an attraction of immune cells toward this tissue due to chronic inflammation.

IgT has been suggested to have particular importance in mucosal surfaces. In fish transiently depleted of IgT, the microbiota could significantly translocate across the epithelium and reach the LP [41]. The key feature of the gut in connection with the immune responses is the considerable number of effector lymphocytes found in the LP, even those in a normal state without disease or inflammation. The present results determine that in Atlantic salmon, the LP is the preferred IgT^+^ B cell location throughout the gut. In teleosts, B cells are considered to mainly be located in the LP and are generally rarely found in the intestinal epithelium [2,31]. The results of the present study confirm these assumptions in Atlantic salmon and showed that, apart from the LP, IgT mRNA^+^ cells were also occasionally found in the stratum granulosum or, less frequently, between both muscle layers, indicating a circulating population that exploits the vascular net associated with physiological diapedesis, as was frequently noticeable in the vaccinated samples.

Similar to the results from Løkka et al. 2014 [13], no significant increase in IgT mRNA^+^ cells in the gut segments could be detected after intraperitoneal vaccination. These results suggest that intraperitoneal injection, a systemic route of immunization, has a low impact on the appearance of IgT^+^ B cells in the gut.

Even though intraperitoneal (IP) administration is the most widely used route of vaccine administration in Atlantic salmon, the role of local B cell responses in the peritoneal cavity needs to be better studied. Upon peritoneal immunization, we have demonstrated the pronounced affluence of IgT and the pIgR mRNA^+^ signal in the peritoneum, which is associated with vaccination adherences and granulomas. Several adjuvants that are used in fish vaccines cause internal organ adhesions or can attach to the parietal peritoneum when injected intraperitoneally [40]. The vaccine included mineral oil (Montanide), and small lesions or cell-vaccine masses could be observed in the abdominal cavity. In the present study, granulomas were noted, as well as angiogenesis in the area of contact with the mesothelium and a melano-macrophage presence, which can be classified as a minor development according to the Speilberg score [42].

In rats, it has been proven that the injection site (into fat or muscle tissue) affects the formation of vaccination granulomas [43] to a substantial extent, with the intra-fat injections being a major predisposition factor. Since salmon is considered a “fatty fish” due to its high fat content, it would be interesting to investigate if vaccination granulomas are as prominent in leaner fish species.

In mammals, damage to peritoneal mesothelial cells is believed to produce adherences by promoting inflammation [44]. The modified morphology of the mesothelium described in the present study may be related to the formation of these adhesions and to be stimulated by the inflammatory environment. In mammals, the peritoneal administration of lipopolysaccharides promotes the migration of peritoneal B1 cells to the milky spots [45,46]; lymphoid cells aggregate in the omentum, where they differentiate into IgM-secreting and IgA-secreting cells, some of which colonize the intestine, nesting in the gut LP [47]. In salmon, there is no phenotype transition and the B cells become activated and mature into antibody-secreting cells [48] where IgM^+^ B cells dominate, according to the previous works mentioned above. However, according to the present results, it has been established that IgT^+^ B cells, along with the attendant pIgR, also showed an active role in fish peritoneal responses that needs to be considered and addressed in future studies. Although migration to the *lamina propria* has not been described, the increase in the IgT transcripts in the peritoneum of the vaccinated fish requires additional research to enhance our understanding of the peritoneum–mucosal axis.

## 5. Conclusions

This study aimed to offer additional insights into the role of IgT in the Atlantic salmon gut. Our results demonstrate that there is a marked and age-influenced difference in the distribution of IgT mRNA^+^ cells throughout the intestines, as the numbers increase with increasing age, but that intraperitoneal vaccination has no detectable impact on the frequency of positive cells. Additionally, we discovered the prominent presence of both IgT and pIgR mRNA in the granulomatous tissue of the peritoneum in the vaccinated fish. Our findings provide further insights into the role of IgT in teleost mucosal immunity post-vaccination.

## Figures and Tables

**Table 1:** Overview of the studied Atlantic salmon groups.

Group 1 (avg. 153 g)		Group 2 (avg. 1717 g)	
Unvaccinated	Vaccinated	Unvaccinated	Vaccinated
(3 fish)	(3 fish)	(3 fish)	(3 fish)

**Table 2:** Target and control probes for RNA in situ hybridization.

	Probe	Accession No.	Target Region (bp)	Catalog No.
Target	IgT	GQ907003	3–883	532171
	pIgR	GQ892057.1	119–1145	845451
Control	*dapB* (negative)	EF191515	414–862	310043
	*ppib* (positive)	NM_001140870	20–934	494421

**Table 3:** IgT and pIgR mRNA^+^ transcript occurrence in the peritoneum of the different studied sections of Atlantic salmon from Group 1 and Group 2, both unvaccinated and vaccinated. Pc: pyloric caeca; FsMi: first section of the medium intestine; SsMi: second section of the medium intestine; Ps: posterior intestine; pIgR: polymeric immunoglobulin receptor.

Probe	Section	Group 1		Group 2	
		Unvaccinated	Vaccinated	Unvaccinated	Vaccinated
IgT	Pc	−	−	−	+
	FsMi	−	−	−	+
	SsMi	−/+	−/+	−/+	++
	Ps	−	−	−	+
pIgR	SsMi	+	−/+	+	−/+

## Data Availability

The datasets generated and analyzed during the current study are not publicly available because, due to the nature of this research, accompanying data to the ones presented herein remain unpublished to date, but are available from the corresponding author on reasonable request.

## Supplementary Materials

The following supporting information can be downloaded at: https://www.mdpi.com/article/10.3390/ani13203191/s1, Figure S1: RNAscope in situ hybridization, demonstrating IgT and pIgR mRNA^+^ distribution in lymphoid organs in Atlantic salmon (*Salmo salar*) as a positive control and *dapB* as a negative control; Figure S2: RNAscope in situ hybridization, demonstrating pIgR mRNA^+^ cells distribution associated with blood vessels in the peritoneal tissue of the second segment of the mid-intestine sections in Atlantic salmon.
