# Supplementary material for: The Distribution of IgT mRNA+ Cells in the Gut of the Atlantic Salmon (Salmo salar L.)
Source: Animals (Basel). 2023 Oct 12;13(20):3191. doi: 10.3390/ani13203191 (PMC10603744; doi:10.3390/ani13203191)
Supplement: Supplementary file 1 [file animals-13-03191-s001.zip › animals-2579456-supplementary.docx]

**
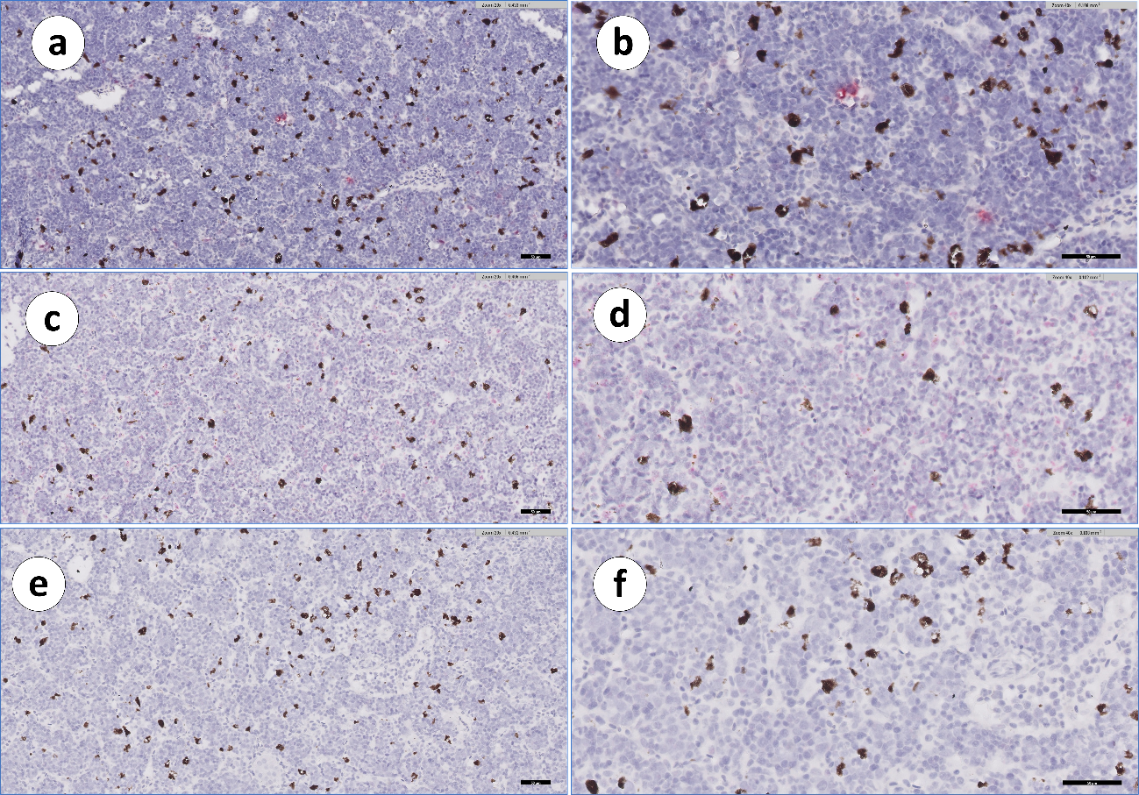
**

**Figure S1**. RNAscope in situ hybridization demonstrating IgT and pIgR mRNA+ distribution in lymphoid organs in Atlantic salmon (Salmo salar) as positive control: (**a**) Head kidney, IgT. (**b**) Head kidney detail, IgT. (**c**) Spleen, pIgR. (**d**) spleen detail, pIgR; and as negative control (**a**) Head kidney, dapB. (**b**) Head kidney detail, dapB. Scale bars 50 μm.

**
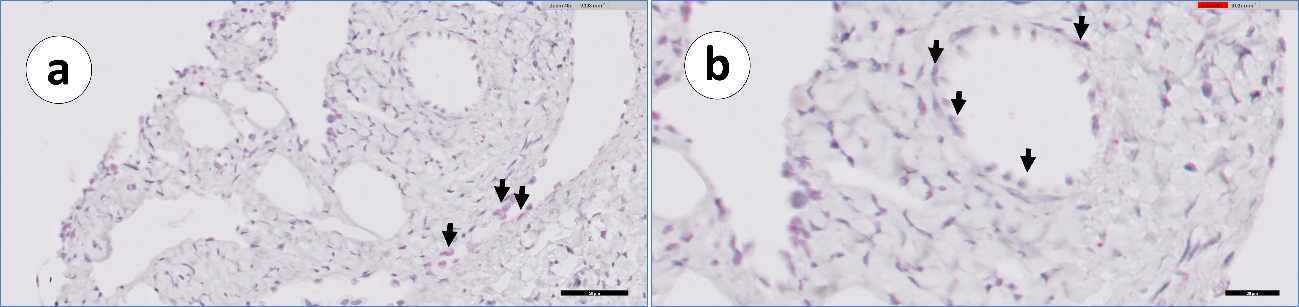
**

**Figure S2.** RNAscope in situ hybridization demonstrating pIgR mRNA^+^ cells distribution associated to blood vessel in the peritoneal tissue of the second segment of the mid-intestine sections in Atlantic salmon. (**a**) Group 2 vaccinated (**b**) detail. Scale bar as follows: (**a**) 50 µm, (**b**) 20 µm.

**Article Information:**

The Distribution of IgT mRNA^+^ Cells in the Gut of the Atlantic Salmon (*Salmo salar* L.)

Pedro Luis Castro ^1,^*, Fran Barac ^2^, Tom Johnny Hansen^3^, Per Gunnar Fjelldal ^3^, Ivar Hordvik ^4^, Håvard Bjørgen ^2^ and Erling Olaf Koppang ^2^

^1^ GIA-ECOAQUA, Universidad de Las Palmas de Gran Canaria, 35001 Telde, Spain

^2^ Unit of Anatomy, Veterinary Faculty, Norwegian University of Life Sciences, 1433 Ås, Norway;
fran.barac@nmbu.no (F.B.); havard.bjorgen@nmbu.no (H.B.); erling.o.koppang@nmbu.no (E.O.K.)

^3^ Matre Research Station, Institute of Marine Research, 5984 Matredal, Norway; tomh@hi.no (T.J.H.);
pergf@hi.no (P.G.F.)

^4^ Institute of Biology, University of Bergen, 5007 Bergen, Norway; ivar.hordvik@uib.no

* Correspondence: pedro.castro@ulpgc.es
